# Gingival Thickness Improvement After Atelocollagen Injection—Retrospective Study

**DOI:** 10.3390/life16010065

**Published:** 2026-01-01

**Authors:** Sylwia Klewin-Steinböck, Anna Duda-Sobczak, Marzena Liliana Wyganowska

**Affiliations:** 1 Department of Periodontology and Oral Mucosa Diseases, Poznan University of Medical Science, 70 Bukowska St., 60-812 Poznan, Poland; 2 Department of Internal Medicine and Diabetology, Poznan University of Medical Science, 2 Mickiewicza St., 60-830 Poznan, Poland; annasobczak@ump.edu.pl

**Keywords:** gingival phenotype, gingival thickness, atelocollagen

## Abstract

**Background:** This study evaluates the increase in gingival thickness following the administration of injectable atelocollagen. **Materials and Methods:** A retrospective analysis was conducted using the medical records of 60 patients with a thin gingival phenotype at baseline, treated between 2017 and 2025. All patients received a standardised protocol for soft tissue thickness modification using atelocollagen injections. Based on the continuation of maintenance therapy, patients were divided into Group A (*n* = 30), consisting of patients who received booster doses at six-month intervals following completion of the full treatment protocol, and Group B (*n* = 30), consisting of patients who did not continue maintenance therapy. The observation period for all patients was five years. Gingival thickness was assessed by periodontal probe transparency using a standard WHO probe (WHO 621) and the Hu-Friedy Colorvue Biotype Probe. Longitudinal changes were analysed using linear mixed-effects models (LMMs) for continuous outcomes and generalised linear mixed-effects models (GLMMs) with a binomial distribution and logit link for binary outcomes, accounting for repeated measurements at the patient level. **Results:** Significant effects of Group and Time, as well as their interaction, were observed for the proportion of sites with a thick gingiva (Group effect: F (1,93.14) = 57.94, *p* < 0.001; Group × Time interaction: *p* < 0.001). GLMM analysis confirmed a significant Group × Time interaction (χ^2^ = 23.11, *p* < 0.001), indicating sustained gingival thickness improvement in Group A and a gradual decrease in effectiveness in Group B. **Conclusions:** Injectable atelocollagen represents a reliable, effective, and user-friendly method for long-term modification of gingival thickness, particularly when supported by maintenance therapy.

## 1. Introduction

Understanding the relationship between soft tissue health and treatment protocols is essential, as clinicians must increasingly address not only patients’ functional needs but also their aesthetic expectations. Predicting treatment outcomes before initiating therapy is, therefore, a critical component of treatment planning and risk assessment.

In 1969, Ochsenbein and Ross [1] introduced the term “gingival biotype” to describe two main types of gingival morphology, which they categorised as pronounced scalloped and flat. Later, in 1986, Claffey and Shanley [2] further characterised tissue biotypes, defining a thin biotype as gingival thickness (GT) less than 1.5 mm and a thick biotype as GT greater than 2 mm. In 1989, Seibert and Lindhe expanded this concept by introducing the term “periodontal biotype”, classifying the periodontium into thin-scalloped and thick–flat forms [3].

During the 2017 World Workshop on the Classification of Periodontal and Peri-Implant Diseases and Conditions [4], the term “periodontal phenotype” was recommended. The workshop emphasised the distinction between biotype and phenotype: whereas the biotype is genetically determined and unalterable, the phenotype reflects observable characteristics that may change over time due to environmental influences or clinical interventions and can vary between sites. The periodontal phenotype comprises two components: the bone morphotype (buccal bone plate thickness) and the gingival phenotype, which includes keratinised tissue width (KT) and gingival thickness (GT).

Gingival thickness can be assessed using several methods, including periodontal probe transparency, transgingival probing, ultrasonography, and cone-beam computed tomography (CBCT) [5,6]. Probe transparency is a simple, reproducible, inexpensive, and non-invasive method that categorises the gingival phenotype based on probe visibility beneath the gingival margin. CBCT provides highly accurate measurements and allows assessment of bone morphology; however, it is associated with higher costs and radiation exposure [7]. Ultrasonography offers radiation-free, non-invasive assessment but requires specialised equipment and operator expertise. Studies have demonstrated minimal differences between ultrasonography and transgingival probing in the anterior region, whereas significant discrepancies have been reported in posterior areas [6]. Transgingival probing remains the gold standard for gingival thickness measurement [8,9].

The 2017 World Workshop recommended evaluating the periodontal phenotype with the aid of a periodontal probe inserted into the sulcus to measure the GT. It is assumed that the probe tip is visible when GT is thin (≤1 mm) and undetectable when GT is thick (>1 mm) [4].

Periodontal surgery enables reconstruction of the gingival tissues for both height and thickness while increasing the width of keratinised gingiva. The subepithelial connective tissue graft (CTG) is considered the gold standard for phenotype modification; however, the greatest improvement in keratinised tissue width has been reported when CTG is combined with an enamel matrix derivative [10,11]. Recent advances in regenerative and aesthetic medicine have introduced minimally invasive, non-surgical approaches for improving gingival thickness, including the use of injectable platelet-rich fibrin (i-PRF), either alone or in combination with microneedling, hyaluronic acid (HA), and collagen-based therapies.

The regenerative potential of platelet concentrates has been recognised since the 1970s due to their growth-factor content [12]. i-PRF promotes tissue repair by releasing high concentrations of growth factors, such as TGF-β, PDGF, VEGF, IGF-1, FGF, and EGF [13] and has been shown to increase gingival thickness [14,15]. However, its clinical use is limited by restricted volume, time-sensitive preparation, and its inability to be sterilised, with an associated risk of contamination [16,17].

Microneedling is a minimally invasive percutaneous technique that induces controlled microinjuries and growth-factor release [18]. In periodontology, it has mainly been studied in combination with i-PRF, with increased keratinized tissue width observed only in combined treatments [19,20].

Hyaluronic acid, a key extracellular matrix component, supports wound healing and tissue regeneration and has demonstrated gingival regenerative potential comparable to i-PRF [21,22].

Collagen is a highly valuable biomaterial for connective tissue regeneration due to its biocompatibility, biodegradability, hydrophilicity, and low immunogenicity. Used in medicine for over 5000 years, collagen is now widely applied in surgery, dentistry, and aesthetic medicine for haemostasis, healing, and tissue remodelling [23,24]. Collagen-based biomaterials support essential regenerative processes such as cell proliferation, differentiation, and neovascularisation.

In aesthetic medicine, bovine collagen was first introduced as a dermal filler in the 1960s, but alternative sources have since been explored for both filling and bio-stimulatory purposes. Fish collagen offers good immunologic compatibility but is sensitive to high temperatures [25,26]. Because 2–4% of individuals are allergic to bovine type I collagen, reducing antigenicity is essential. Atelocollagen, produced by removing the N- and C-terminal telopeptides responsible for most antigenicity, shows low immunogenicity and is considered a safe biomaterial [27].

Currently, porcine and equine collagens are most used. Equine collagen has a more loosely organised structure, and its production involves minimal chemical modification aside from telopeptide removal. Micronisation further improves atelocollagen’s dissolution, bioavailability, and tissue absorption.

Injectable collagen has recently been introduced as a connective tissue biostimulator. It is widely used in orthopaedics for non-surgical management of tendon and ligament injuries [28,29] and in aesthetic medicine for skin bio-rejuvenation [30,31]. Due to the structural and functional similarities between skin and oral mucosa, injectable atelocollagen has been introduced into dentistry, although long-term clinical data remain limited. Linerase^®^, the material used in this study, promotes fibroblast proliferation, extracellular matrix synthesis (including new collagen fibre formation), and fibroblast-mediated angiogenesis. Unlike i-PRF, atelocollagen does not require blood collection and involves a significantly shorter preparation time.

Our previous studies demonstrated promising outcomes in non-surgical treatment of gingival tissues, with statistically significant improvements in gingival recession depth, papillary height, and gingival thickness following two or three collagen injections [32]. Additionally, atelocollagen was successfully applied in patients with Hashimoto’s disease, resulting in reduced gingival bleeding [33]. However, these studies were limited by small sample sizes and short follow-up periods.

The aim of the present study was to evaluate changes in gingival thickness over a 5-year follow-up period following injections of type I atelocollagen based on data obtained from patients’ medical records.

## 2. Materials and Methods

This study was conducted in accordance with the ethical principles of the Declaration of Helsinki and was approved by the Bioethics Committee of Poznan University of Medical Science (Resolution No. 919/16 of 15 September 2016 and 150/17 of 02 February 2017). Informed consent was obtained from all participants. Informed consent for publication was obtained from the patients.

### 2.1. Participants

The study results were obtained through the retrospective analysis of data from patients’ medical records. Data of 60 patients (both male and female) with a thin gingival phenotype treated at the Department of Periodontology and Oral Mucosa Diseases, Poznan University of Medical Sciences, between 2017 and 2025, were collected. All patients underwent full protocol treatment according to the standard protocol for soft tissue phenotype modification—atelocollagen injections—available at the Department. Patients were classified into the retrospective study according to treatment continuance.

At the beginning of the study, patients were between 27 and 60 years of age. According to the protocol, all included patients presented clinically healthy gingiva, absence of periodontitis, probing pocket depth (PPD) ≤ 3 mm, bleeding on probing (BOP) < 10%, and well-maintained oral hygiene.

### 2.2. Description of Standard Treatment Protocol Used in the Department

The inclusion criteria for treatment are a thin gingival phenotype and the presence of keratinised gingiva, corresponding to Miller Class I or Cairo Class C1 recession defects.

The exclusion criteria for the treatment protocol included active orthodontic treatment, dental caries, a history of periodontal surgery in the relevant area, smoking, pregnancy or breastfeeding, diabetes, immunosuppressive therapy, anticoagulant treatment, and neoplastic diseases. Additional health conditions, such as liver disease, coagulation disorders, and anaemia, are also grounds for exclusion from the treatment. Two weeks before starting treatment, patients completed an intra-oral examination and received hygiene instructions and, if necessary, plaque or calculus removal.

Gingival phenotype is assessed according to the recommendations of the American Academy of Periodontology and the European Federation of Periodontology using periodontal probe transparency with a standard WHO probe (WHO 621) and Hu-Friedy Colorvue biotype probes. Probe visibility with the WHO probe classified the gingiva as thin (visible) or thick (non-visible). The Colorvue probe is used sequentially (white, green, blue) to classify gingival thickness as thin, medium, thick, or very thick. Measurements are performed around the first or second molar, canine, and first incisor in each quadrant.

**Figure 1:**
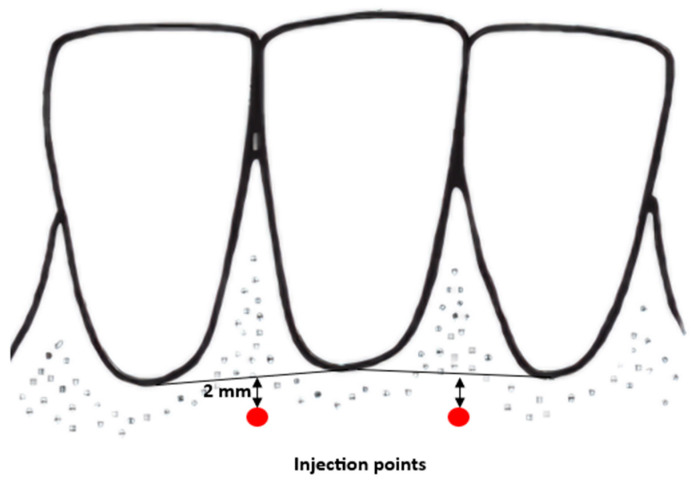
Points of injection diagram.

**Figure 2:**
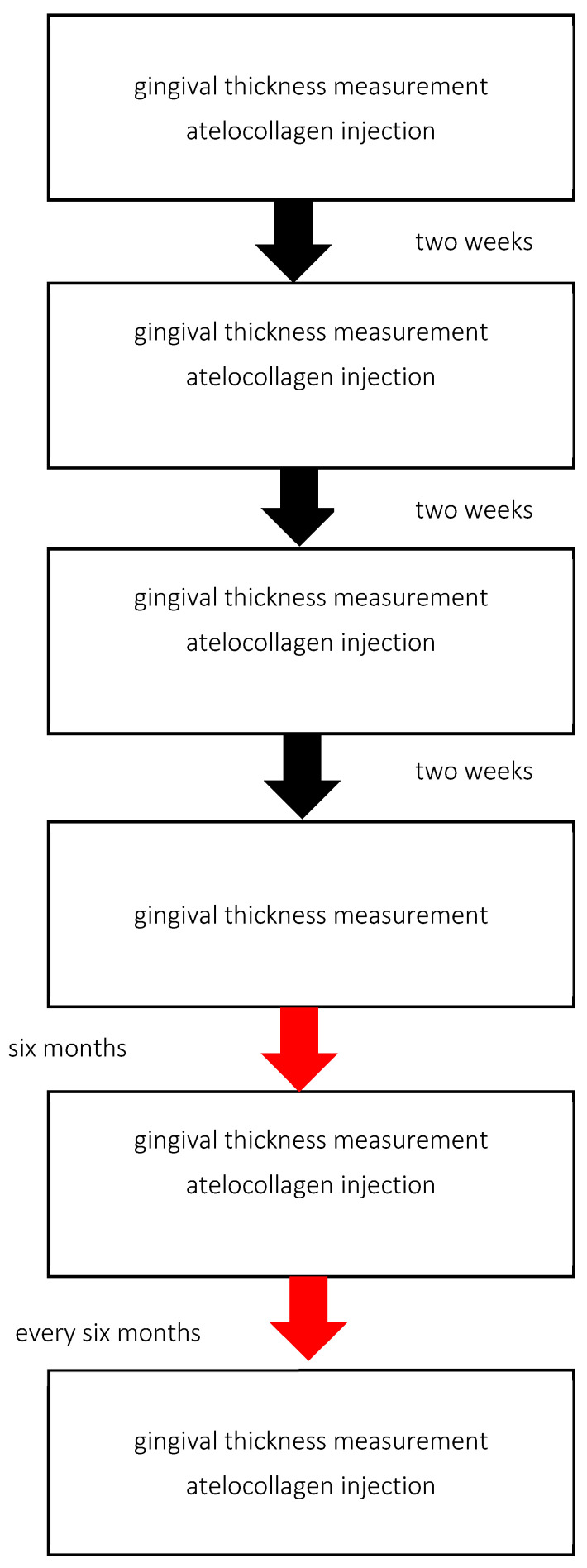
Treatments based on standard protocol design.

During the visit, patients receive injections of atelocollagen into the vestibular side of the alveolar process. In all cases, lyophilised, micronised type I equine atelocollagen (Linerase, CSD S.r.I (Italy), distributed by BD Aesthic, Warsaw, Poland^®^, 100 mg), diluted in 5 mL of 0.9% NaCl, is used. Linerase^®^ is a class III medical device (CE 0477 EPT 0477.MDD.21/4439 EPT 0477.MDD.22/GP0047). A volume of 0.05 mL is injected per point into the keratinised gingiva approximately 2 mm above the base of the gingival papilla across the full arch, in three sessions at two-week intervals. Injection points are spaced approximately 10 mm apart (range: 7–13 mm) (Figure 1). Injections are performed using 1 mL syringes and 30-gauge, 4 mm needles. Gingival thickness is assessed at each visit and two weeks after the third injection. A subset of patients continued maintenance therapy with atelocollagen administered every six months (Figure 2).

### 2.3. Study Description

**Figure 3:**
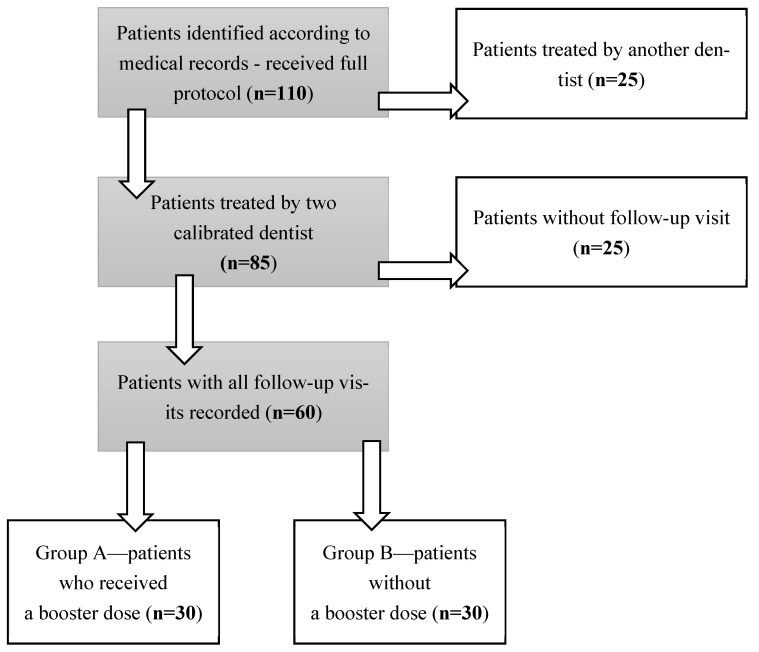
Study design.

A structured search of medical records was conducted (Figure 3). 112 patients who received the full protocol were identified. From this group, 25 medical records of patients treated by physicians other than the two calibrated clinicians were excluded. From the remaining records, another 25 were excluded due to the lack of regular 6-month follow-up visits. The remaining 60 records were divided into two groups: A and B, according to treatment protocol design (Figure 3). Group A included 30 patients who received a booster dose (one application) every six months after completing the full protocol, for a period of five years. Group B consisted of patients who did not continue therapy after completing the full protocol. All patients remained under the department’s care and attended regular follow-up visits. The gingival thickness and oral hygiene were assessed every six months.

### 2.4. Statistical Analysis

All analyses were performed in R (version 4.2.5; R Foundation for Statistical Computing, Vienna, Austria). Two outcomes were analysed: (1) the proportion of sites classified as having a thick gingival phenotype (StandardProbe_percent) and (2) a binary classification of gingival biotype thickness (Hu-FriedyProbes).

Time was represented in two ways. For the continuous outcome (StandardProbe_percent), time was coded as a 12-level categorical factor (time levels 0, 2, 4, 6, 12, 18, 24, 30, 36, 42, 48, 54 months) in the linear mixed-effects model. For the binary outcome (Hu-FriedyProbes), time was coded as a continuous numeric variable corresponding to the same assessment schedule (0–54 months) in the generalised linear mixed-effects model.

Changes in StandardProbe_percent were analysed using a linear mixed-effects model (LMM) with Group (A vs. B), Time, and their interaction (Group × Time) specified as fixed effects. Participant ID was included as a random intercept to account for within-subject repeated measures. Degrees of freedom were estimated using the Satterthwaite approximation. Estimated marginal means (EMMs) with 95% confidence intervals were computed to quantify group differences at clinically relevant timepoints (baseline, 6, 12 months, and 54 months).

For the binary Hu-FriedyProbes outcome, a generalised linear mixed-effects model (GLMM) with a binomial distribution and logit link was fitted, using the same fixed- and random-effects structure as above. Model parameters were estimated Via maximum likelihood with the Laplace approximation. Wald z-tests were used to evaluate individual fixed effects, and Wald Type II Chi-square tests were used to assess the significance of Group, Time, and Group × Time terms. The effect of Time was statistically significant (χ^2^(1) = 8.40, *p* < 0.01), and the Group × Time interaction was also significant (χ^2^(1) = 23.11, *p* < 0.001), indicating divergent temporal trajectories between groups. The baseline difference between groups was not significant (χ^2^(1) = 0.05, *p* = 0.82).

As a sensitivity consideration, it is acknowledged that unmeasured biological or behavioural factors (e.g., individual wound-healing capacity, subtle oral hygiene differences) may influence long-term gingival thickness changes and could not be fully controlled in the present design.

Model-based predicted probabilities with 95% confidence intervals were visualised using marginal effects plots.

## 3. Results

### 3.1. Demographic Analysis

The demographic characteristics of the participants are shown in Table 1. Predominance of female participants was observed (in total, in group A and in group B).

### 3.2. Gingival Thickness Analysis

A total of 60 participants completed the study (30 in group A and 30 in group B). Baseline characteristics, including gingival phenotype distribution, did not differ significantly between groups.

A linear mixed-effects model demonstrated significant main effects of Group and Time, as well as a significant Group × Time interaction for the proportion of sites exhibiting a thick gingiva (StandardProbe_percent). The effect of Group (A vs. B) was statistically significant (F(1, 93.14) = 57.94, *p* < 0.001), indicating consistently higher proportions of thick gingiva sites in Group A compared with Group B. A significant effect of Time was also observed (F(10, 640.15) = 361.36, *p* < 0.001), reflecting dynamic changes in gingival thickness over the follow-up period.

**Figure 4:**
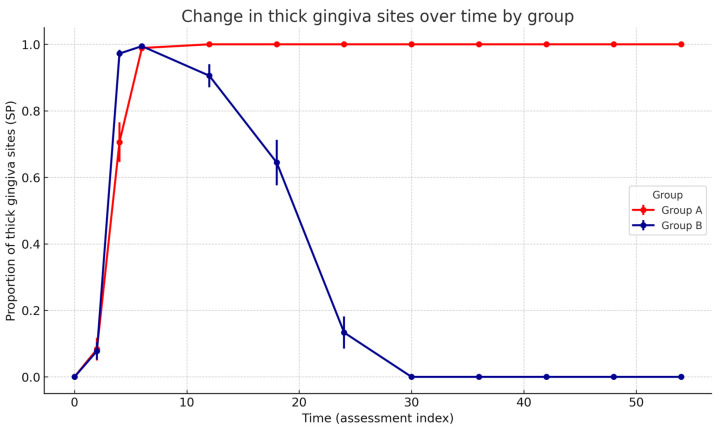
Change in the proportion of sites with a thick gingiva (Standard Probe measurement) over time in A and B groups. Points show model-free group means at each assessment index with SEM error bars; lines connect successive time points. The statistical note summarises the GLMM (binomial, logit) results: significant Group × Time interaction (*p* < 0.001), significant decreasing Time effect in Group B (*p* < 0.001), and no baseline group difference (*p* = 0.82).

Critically, the Group × Time interaction was highly significant (F(10, 640.15) = 419.46, *p* < 0.001), demonstrating distinct temporal trajectories between groups. Estimated marginal means (EMMs) confirmed the divergent long-term trajectories between groups. At baseline (0 months), the proportion of sites with a thick gingiva was comparable in both groups (Group A: 0.00, 95% CI −0.05 to 0.05; Group B: 0.00, 95% CI −0.05 to 0.05). After 6 months, both groups demonstrated a marked increase (Group A: 0.99, 95% CI 0.94 to 1.04; Group B: 0.99, 95% CI 0.95 to 1.04). At 12 months, stabilisation was observed in Group A (1.00, 95% CI 0.95 to 1.05), whereas a decline had already begun in Group B (0.90, 95% CI 0.86 to 0.95). At the final follow-up (54 months), Group A maintained the treatment effect (1.00, 95% CI 0.95 to 1.05), while Group B returned to baseline levels (0.00, 95% CI −0.05 to 0.05). Estimated marginal means across all time points are presented in Figure 4.

**Figure 5:**
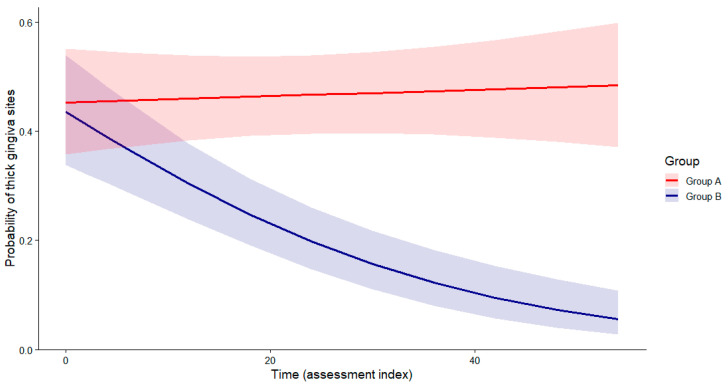
Predicted probability of thick gingiva sites over time (Hu-Friedy Colorvue biotype probes). The B group shows a progressive decline, while the A group remains stable. Group × Time interaction (Wald χ^2^ test), *p* < 0.001).

For the binary phenotype classification (Hu-FriedyProbes), a generalised linear mixed model with a binomial distribution and logit link was fitted. There was no significant baseline difference between groups (*p* = 0.82, Wald z-test). A significant negative effect of Time was observed (β = −0.048, SE ≈ 0.008, *p* < 0.001, Wald z-test), indicating a progressive decrease in the probability of a gingival thickness in Group B. The Group × Time interaction was significant (β = 0.050, SE ≈ 0.010, *p* < 0.001, Wald z-test), demonstrating stabilisation or a slight increase in Group A over time (Table 2). Model-based predicted probabilities with 95% confidence intervals confirmed a progressive decline in Group B and long-term stabilisation in Group A (Figure 5).

Model assumptions were evaluated for both the LMM and GLMM. For the LMM, residual plots showed no major deviations from normality or homoscedasticity. For the GLMM, checks for overdispersion and influential observations indicated no model misspecification. Overall, diagnostics supported the adequacy of both models.

As a sensitivity analysis, the primary models were re-estimated using alternative time coding schemes (time treated as categorical rather than continuous) and restricted datasets excluding participants with incomplete follow-up measurements. Across all specifications, the direction and significance of the Group × Time interaction remained unchanged, supporting the robustness of the findings. Nonetheless, because this was an observational longitudinal study, the potential influence of unmeasured confounding (e.g., unreported behavioural changes or local anatomical variation) cannot be fully excluded.

There was no significant baseline difference between Group A and Group B. Time was associated with a significant decrease in the probability of a thick gingiva in Group B. A significant Group × Time interaction indicates that this decreasing trend was attenuated in Group A, where the probability remained stable or increased slightly over time.

## 4. Discussion

Atelocollagen has been widely investigated as a biomaterial in regenerative medicine, with applications in tissue engineering, wound healing, and soft-tissue regeneration. Experimental evidence indicated that atelocollagen can stimulate collagen synthesis at the injection site, contributing to improved tissue structure and consistency. In senescent human fibroblasts and aged murine skin, atelocollagen has been shown to increase GlyT1 expression, accompanied by reduced oxidative stress and decreased expression of MMP-1, MMP-3, and MMP-9 [34]. Concurrently, restoration of type I and III collagen production was observed, resulting in increased collagen fibre density and improved skin elasticity [34]. Injected atelocollagen is expected to undergo enzymatic degradation within the tissues, resulting in the gradual formation of smaller collagen fragments, peptides, and amino acids [35]. These degradation products may subsequently influence resident cells through established pathways involved in extracellular matrix turnover and tissue remodelling [36,37]. Amino acids and tripeptides play an important role in stimulating fibroblasts and enhancing collagen production [34]. Collagen peptides and hydrolysates exhibit superior solubility, diffusion capacity, and bioavailability [38]. As fundamental structural components of the extracellular matrix (ECM), they provide the essential substrates required for collagen fibre assembly. Beyond their structural function, many amino acids and bioactive peptides act as signalling molecules capable of modulating fibroblast metabolism, proliferation, and migration. Tripeptides can act as signalling molecules that activate fibroblast pathways responsible for increased collagen gene expression and extracellular matrix remodelling [39]. Several studies have demonstrated that small peptides promote fibroblast proliferation, migration, and adhesion, which are key processes in tissue regeneration [36,38]. Moreover, these peptides have been shown to enhance collagen and elastin synthesis while simultaneously reducing the expression of matrix metalloproteinases (e.g., MMP-1 and MMP-3), thereby limiting collagen degradation and supporting the stability of newly formed tissue [40].

Most investigations to date have focused on dermal fibroblasts, and long-term clinical data on injectable atelocollagen therapy in dentistry remain limited. Nevertheless, similar biological mechanisms are likely applicable to gingival tissues, given the comparable fibroblast-mediated organisation of the extracellular matrix. The high metabolic activity of gingival fibroblasts may further enhance the tissue response to collagen-derived peptides, thereby contributing to the observed improvement and stabilisation of the gingival phenotype following atelocollagen therapy. Short-term studies of tropocollagen injections have demonstrated a significant reduction in tooth hypersensitivity and a potential increase in gingival thickness in orthodontically treated patients [41]. Additionally, in vitro studies on human gingival fibroblasts (hGFs) have shown increased cell viability following exposure to injectable collagen [42].

Following injectable atelocollagen therapy, the newly synthesised collagen within the gingiva is subject to continuous turnover mediated by fibroblasts. Fibroblasts are capable of internalising and degrading collagen via receptor-mediated pathways, contributing to active extracellular matrix remodelling. More specifically, the balance between collagen synthesis and degradation is finely regulated. Collagen type I, the predominant collagen in gingival connective tissue, is subject to both synthesis and breakdown by matrix metalloproteinases (MMPs) and fibroblast-mediated phagocytosis [43]. Interestingly, clinically observed gingival phenotype improvement is not permanently fixed. Over time, the endogenous physiological processes of the gingival fibroblasts [44] (matrix turnover and remodelling) may gradually degrade and reorganise the newly deposited collagen, leading to a partial or complete regression of the phenotype change [45]. This physiological turnover may underscore why, despite initial gains in gingival thickness, maintenance therapy is required to preserve clinical results in the long term.

After completion of the full atelocollagen injection protocol (three injections), the present study demonstrated a consistent improvement in the gingival phenotype in all patients. A uniform transition from a thin to a thicker phenotype was observed, indicating that the protocol provides predictable and clinically meaningful soft-tissue enhancement. These findings suggest that atelocollagen injections effectively stimulate gingival tissue remodelling and may represent a biologically driven alternative to more invasive mucogingival procedures [32].

An important aspect of these findings relates to the durability of the treatment effect. In Group A, where patients continued treatment with a booster dose, the achieved gingival thickness remained stable throughout the entire observation period. The additional booster dose did not induce further thickening of the gingiva in places where the full protocol was performed. Notably, no cases of gingival overgrowth were observed. Assessment with Hu-Friedy (HF) biotype probes confirmed that once the tissue reached the “thick” category after three injections, it consistently remained within this range despite additional booster therapy. Similarly, when the gingival tissue reached the “medium thick” category, it remained stable, and this condition was maintained for the full duration of follow-up.

In contrast, patients in Group B, who did not receive maintenance therapy, exhibited a gradual reduction in gingival thickness over time, ultimately returning to their baseline phenotype. Although the timing of phenotype regression varied among individuals, early signs of reversal were typically observed approximately one year after completion of the protocol. In all patients, a complete return to the initial thin phenotype occurred no later than two and a half years after treatment (Figure 4).

Patients treated according to the therapeutic protocol did not report any adverse side effects. No local or systemic complications were reported during the procedure or throughout the follow-up period. Specifically, there were no reports of excessive pain, prolonged swelling, bleeding, infection, allergic reactions, tissue necrosis, or signs of gingival overgrowth at the injection sites. The treatment was well tolerated by all patients. Patients reported a reduction or complete resolution of tooth hypersensitivity after therapy. This improvement was associated with gingival tissue gain not only in thickness but also in vertical height, resulting in partial coverage of previously exposed root surfaces.

Our previous in vitro study indicated that Linerase^®^ does not suppress apoptosis in gingival fibroblasts. In fact, the highest number of apoptotic cells was observed on the first day after atelocollagen stimulation, suggesting an early cellular stress response prior to subsequent normalisation and remodelling [46]. It is important to note that in the present study, none of the patients exhibited gingival tissue overgrowth at any stage of the study, which confirms the biological safety and controlled action of atelocollagen within the gingival tissues. This observation is particularly relevant in the context of therapies aimed at modifying the gingival phenotype, as excessive or uncontrolled tissue proliferation could compromise clinical outcomes or lead to aesthetic concerns.

Importantly, the procedure proved not only effective but also easy to perform and well-tolerated by patients, making it a practical option in routine periodontal and mucogingival therapy. The repeated applications of atelocollagen contributed to the stabilisation and thickening of the gingival phenotype, which is clinically relevant given the protective role of a thicker gingival biotype in preventing soft-tissue recession, improving aesthetic outcomes, and enhancing long-term stability following periodontal or implant procedures.

It is also worth noting that we did not directly adopt the protocols used in aesthetic medicine. Due to the higher activity of gingival fibroblasts and the distinct metabolic characteristics of oral soft tissues, we modified the procedure. Instead of the standard protocol used in aesthetic medicine, we implemented a protocol consisting of three initial injections followed by one booster injection every six months [32]. This adjustment reflects the unique biological properties of gingival tissues and provides a tailored approach optimised for intraoral application.

The proposed protocol represents our original approach, specifically designed for patients with a thin gingival phenotype. Its applicability may vary across countries depending on the availability of materials. Overall, our findings confirm that atelocollagen injections represent a reliable, effective, and user-friendly method for gingival phenotype modification, with promising implications for both periodontal health and aesthetic treatment planning.

## 5. Conclusions

The rapid development of regenerative medicine and the adaptation of techniques and materials used in aesthetic medicine have offered new non-surgical possibilities for improving the biotype of the gingiva. The observed improvements in gingival phenotype after atelocollagen injections support the concept that minimally invasive biologic therapies can modify gingival tissue characteristics without the need for surgical intervention. This aligns with recent trends in periodontal regeneration, emphasising biomaterials that stimulate tissue remodelling while maintaining high patient comfort.

Overall, our findings confirm that atelocollagen injections represent a reliable, effective, and user-friendly approach for gingival phenotype modification, with potential benefits for both periodontal health and aesthetic outcomes. The use of atelocollagen can help to bypass or delay the need for periodontal surgery.

## Figures and Tables

**Table 1:** Demographic characteristics of the participants. Mann–Whitney U test. * chi-square test.

Variables	Group A		Group B	*p*-Value
**Age range**	N (%)		N (%)	
**≤29**	11 (37)		7 (23)	
**30–39**	8 (27)		8 (27)	
**40–49**	6 (20)		9 (30)	
**50–59**	4 (13)		5 (17)	
**≥60**	1 (3)		1 (3)	
**Median (IQR)**	33 (28–44)		39.5 (30–48)	0.2
**Gender**				
**Male/Female**	8 (27)/22 (73)		10 (33)/20 (67)	0.6 *

**Table 2:** Results of the generalised linear mixed-effects model (GLMM) assessing the probability of exhibiting a thick gingiva over time. The model included Group (A vs. B), Time (continuous assessment index), and the Group × Time interaction as fixed effects, with participant ID modelled as a random intercept. Odds ratios (OR) with 95% confidence intervals (CI) and *p*-values from Wald Type II Chi-square tests are presented.

Parameter	OR	95% CI (OR)	*p*-Value
**Group (A vs. B)**	1.07	0.60–1.92	0.818
**Time (per assessment unit)**	0.95	0.94–0.97	<0.001
**Group × Time**	1.05	1.03–1.07	<0.001

## Data Availability

The datasets used and analysed during the current study are available from the corresponding author upon reasonable request.

## Abbreviations

TGF- β	transforming growth factor-β
IGF-1	insulin-like growth factor-1
PDGF	platelet-derived growth factor (PDGF)
VEGF	vascular endothelial growth factor
FGF	fibroblast growth factor
EGF	epidermal growth factor (EGF)
PDEGF	platelet-derived epidermal growth factor
